# Emerging Treatment Approaches for Parkinson’s Disease

**DOI:** 10.3389/fnins.2018.00693

**Published:** 2018-10-08

**Authors:** Thomas B. Stoker, Kelli M. Torsney, Roger A. Barker

**Affiliations:** ^1^John van Geest Centre for Brain Repair, Department of Clinical Neurosciences, University of Cambridge, Cambridge, United Kingdom; ^2^Wellcome Trust – Medical Research Council Stem Cell Institute, University of Cambridge, Cambridge, United Kingdom; ^3^Department of Neurology, Cambridge University Hospitals NHS Foundation Trust, Cambridge, United Kingdom; ^4^Department of Medicine for the Elderly, Cambridge University Hospitals NHS Foundation Trust, Cambridge, United Kingdom

**Keywords:** α-synuclein, novel therapies, Parkinson’s disease, regenerative therapies, stem cells

## Abstract

Parkinson’s disease (PD) is the second most common neurodegenerative disease, manifesting as a characteristic movement disorder with a number of additional non-motor features. The pathological hallmark of PD is the presence of intra-neuronal aggregates of α-synuclein (Lewy bodies). The movement disorder of PD occurs largely due to loss of dopaminergic neurons of the substantia nigra, resulting in striatal dopamine depletion. There are currently no proven disease modifying treatments for PD, with management options consisting mainly of dopaminergic drugs, and in a limited number of patients, deep brain stimulation. Long-term use of established dopaminergic therapies for PD results in significant adverse effects, and there is therefore a requirement to develop better means of restoring striatal dopamine, as well as treatments that are able to slow progression of the disease. A number of exciting treatments have yielded promising results in pre-clinical and early clinical trials, and it now seems likely that the landscape for the management of PD will change dramatically in the short to medium term future. Here, we discuss the promising regenerative cell-based and gene therapies, designed to treat the dopaminergic aspects of PD whilst limiting adverse effects, as well as novel approaches to reducing α-synuclein pathology.

## Introduction

Parkinson’s disease (PD) is the second most common neurodegenerative disease, typically manifesting with a characteristic movement disorder, consisting of bradykinesia, rigidity, rest tremor and postural instability (Kalia and Lang, 2015). Additional non-motor manifestations occur, including depression, anxiety, sleep abnormalities, constipation and cognitive decline with dementia, which can significantly impair the patient’s quality of life (Khoo et al., 2013).

Pathologically, PD is characterized by the presence of abnormal intra-neuronal aggregates of α-synuclein, termed Lewy bodies and Lewy neurites (Spillantini et al., 1997). Whilst the mechanistic role of Lewy bodies is not fully understood, α-synuclein is clearly central to the pathogenesis of PD, as is highlighted by the fact that rare *SNCA* mutations, duplications, or triplications cause autosomal dominant familial PD (Klein and Westenberger, 2012). The movement disorder of PD occurs in part due to the selective loss of dopaminergic neurons of the substantia nigra pars compacta, resulting in depletion of dopamine in the striatum, whilst non-motor manifestations predominantly occur due to more widespread neurodegeneration, affecting the cortex and a number of brainstem regions (Selikhova et al., 2009; Kalia and Lang, 2015). Dopaminergic loss also has wider effects, including on sleep and cognition (Williams-Gray et al., 2009).

Since the introduction of levodopa in the 1960s, there have been relatively few developments in the treatment of PD. There are no disease-modifying treatments, and the chronic use of levodopa results in significant adverse effects, which themselves constitute an important part of advanced PD (Jenner, 2003; Kalia and Lang, 2015). However, a number of exciting treatment approaches are either already in, or will soon begin in clinical trials, and the landscape of PD treatment is likely to change dramatically over the coming decades. In this review, we discuss the emerging treatment approaches, and the form that future PD management might take in the next few years.

## Current Treatment Options for Parkinson’s Disease

There are currently no disease-modifying treatments for PD, and management predominantly consists of dopaminergic drugs. The most commonly used of these are preparations of levodopa, the precursor of dopamine, which is administered in combination with a dopa-decarboxylase inhibitor which acts to limit some of the side effects, such as nausea. Dopamine agonists, such as ropinirole or rotigotine, are also used. Monoamine oxidase B inhibitors, such as rasagiline and selegiline, and catechol-O-methyltransferase (COMT) inhibitors such as entacapone, can be used to reduce the metabolism of endogenous dopamine. These treatments can restore dopaminergic activity in the striatum, heralding improvements in the motor features of PD. However, they do not treat many of the non-motor features, which are particularly disabling for many patients. Indeed, in some cases treatments may exacerbate some of the non-motor symptoms, such as postural hypotension and neuropsychiatric problems (Young et al., 1997; Kujawa et al., 2000).

Whilst these treatments can cause dramatic improvements in the motor features of PD, especially in the early stages, prolonged use of levodopa in particular results in significant adverse effects, which form an important part of the clinical picture in advanced PD. The non-physiological continuous delivery of dopamine to the striatum is thought to underlie the problematic dyskinesias (abnormal involuntary jerky movements) (Jenner, 2003; Huot et al., 2013), and significant fluctuations in motor function can occur due to erratic absorption of the drug and variable transit of levodopa into the brain – giving the so called on-off phenomenon (Nutt et al., 1984). These medications also result in off-target effects, resulting from their delivery to areas of the brain other than the striatum, which is thought to be the basis for the neuropsychiatric adverse effects that can occur, including hallucinations and impulse control disorder (Ernst, 1969; Voon et al., 2009).

Other treatment options include deep brain stimulation (DBS), which can be very effective in controlling the movement disorder of PD, but like the dopaminergic medications it does not help with most of the non-motor manifestations (Kalia et al., 2013). Though DBS is a safe treatment approach, there are other potentially problematic adverse effects including speech dysfunction and psychiatric disturbance, as well as the general risks associated with a neurosurgical procedure, and this treatment is only suitable in a minority of PD cases (Benabid, 2003).

One approach to delivering dopamine in a more physiological manner is the use of levodopa-intestinal gel, which results in a more predictable release of dopamine than is possible with oral preparations. This may be useful in reducing the motor adverse effects of dopaminergic treatment, but is currently very expensive for widespread use, and is not without complications given the surgery that is necessary for its placement (Olanow et al., 2014). In addition, as with DBS and apomorphine pumps, patients are attached to a device which must be worn all of the time, which is undesirable for many individuals.

So even though there are effective treatment options for the motor features of PD, these come with significant problems, and none of them are able to slow the progression of disease, or improve the disabling non-motor features. Indeed some of these non-motor features are even partly driven by these drugs. There is therefore a need to identify novel methods of restoring striatal dopamine in a targeted and physiological manner, as well as a need to identify treatments that are able to prevent ongoing neurodegeneration and progression of disease. Several experimental approaches are currently being investigated in pre-clinical studies and clinical trials, and it seems likely that the treatment of PD will see dramatic changes in the coming decades. Here, we discuss some of the most promising prospective treatment approaches.

## Restoration of Dopaminergic Deficits

Treatment of many of the motor symptoms of PD can be achieved through restoration of striatal dopaminergic tone. This may be accomplished through targeted delivery of dopamine-producing cells, or the use of viruses to deliver genes encoding the enzymes required for dopamine biosynthesis into the striatum. Targeting these regenerative treatments to the striatum, the site of greatest dopamine loss in PD, would minimize the off-target effects seen with oral dopamine-replacement.

### Cell-Based Approaches

A variety of cell sources have now been investigated for transplantation in PD, with varying degrees of success (**Table 1**; Barker et al., 2015). The use of human fetal ventral mesencephalon (VM) tissue grafts in humans has provided proof-of-concept that such cell-based approaches can be effective in treating many of the critical features of PD, but ethical and logistical barriers (chiefly the unpredictable and inadequate supply of fetal tissue) means that this approach will never be a viable mainline therapy for this condition (Barker et al., 2015). What is required, is a renewable source of dopamine-producing cells, or their progenitors, that have the ability to integrate into the host brain, extend axons over adequate distances to innervate the whole striatum, and survive within the host for years. These criteria are most likely to be met by stem cell-derived neurons, which now offer the most likely approach to delivering a clinically useful and scalable cell-based therapy for PD (Barker et al., 2018).

**Table 1:** Experimental regenerative approaches to treating Parkinson’s disease.

Approach	Trialed in humans?	Major limitations	Reference
**Cell-based treatments**			
Adrenal medullary tissue	Yes	Poor graft survival Neuropsychiatric complications	Backlund et al., 1985; Lindvall et al., 1987; Madrazo et al., 1987; Jiao et al., 1988; Drucker-Colín et al., 1988; Kelly et al., 1989; Allen et al., 1989; Goetz et al., 1990
Human fetal ventral mesencephalon	Yes	Unpredictable supply of tissue Ethical barriers	Lindvall et al., 1989; Lindvall et al., 1990; Redmond et al., 1993; Lindvall et al., 1994; Freeman et al., 1995; Kordower et al., 1995; Wenning et al., 1997; Brundin et al., 2000; Mendez et al., 2000; Freed et al., 2001; Stover et al., 2005; Gross et al., 2011;
Porcine ventral mesencephalon	Yes	Poor graft survival Little clinical benefit	Schumacher et al., 2000
Retinal pigment epithelium cells	Yes	Little clinical benefit	Stover et al., 2005; Gross et al., 2011;
Carotid body cells	Yes	Little clinical benefit	Arjona et al., 2003; Mínguez-Castellanos et al., 2007
ESC-derived neural progenitors	No	Ethical barriers Theoretical risk of tumorigenesis Requirement for immunosuppression	
iPSC-derived neural progenitors	No	Theoretical risk of tumorigenesis Heterogeneity in cell product between individuals Regulatory challenges and cost	
Mesenchymal stem cells	Yes	Not possible to generate authentic dopaminergic neurons Theoretical risk of tumorigenesis	Venkataramana et al., 2010
**Virus-mediated gene delivery**			
AAV (AADC)AAV (neurturin)	YesYes	Limited cargo-size Little clinical benefit in randomized trials	Christine et al., 2009; Muramatsu et al., 2010 Marks et al., 2008
AAV (glutamic acid decarboxylase)	Yes		LeWitt et al., 2011
Lentivirus (AADC, cyclohydrolase, tyrosine hydroxylase)	Yes	Theoretical risk of insertional mutagenesis	Palfi et al., 2014

Trials of embryonic stem cell (ESC)-derived and induced pluripotent stem cell (iPSC)-derived dopaminergic neuron progenitors are due to commence over the next one or 2 years (Barker et al., 2017). Details on design of these trials, including cell-delivery method, patient-selection criteria, immunosuppression, and study end-points are discussed in detail elsewhere (Barker et al., 2017). ESC lines are isolated from the early blastocyst, and have been generated from surplus human embryos derived from *in vitro* fertilization procedures (Thomson et al., 1998). It is now possible for these to be reprogrammed to midbrain dopaminergic neuronal progenitors with high efficiency, which have been safely grafted into rodents, with restoration of motor deficits (Kriks et al., 2011; Grealish et al., 2014). As with the prior human fetal mesencephalon grafts, these cells would be allogenic, so would likely require a period of immunosuppression, which is associated with risks to the host, including infection and malignancy (Gutierrez-Dalmau and Campistol, 2007). There are of course ethical considerations with the use of embryonic tissue, but in most cultures this is probably considered acceptable in comparison to the use of fetal tissue (Barker and de Beaufort, 2013).

In contrast, iPSCs are derived through the reprogramming of somatic cells such as fibroblasts, into pluripotent cells, which can subsequently be converted into dopaminergic neuron progenitors (Takahashi et al., 2007; Kikuchi et al., 2017). These have been shown to integrate into the host striatum in non-human primates, yielding motor improvement with no tumor formation at 2 years (Kikuchi et al., 2017). This concept is appealing, as it could potentially provide autologous tissue grafts, which may circumvent the need for immunosuppressive agents. One disadvantage of using autologous cells, however, is that the grafted product will contain any genetic PD-susceptibility factors that contributed to the development of PD in the host. Acquisition of PD pathology has been seen in allogeneic fetal VM grafts, and it may be that this occurs more rapidly in cells with an inherent genetic predisposition to this type of pathology, meaning that the benefit of iPSC-derived grafts may be lost sooner in comparison to ESC-derived grafts (Li et al., 2016). Whilst ESC reprogramming can be highly reproducible, providing a consistent cell product for the use in a large population of patients, there is a high degree of variability between individuals in the reprogramming of adult fibroblasts into iPSCs, and subsequent differentiation to neural cells. This poses regulatory challenges, as each iPSC line derived would essentially be a different product and would potentially be subject to regulatory approval and extensive preclinical testing which brings with it substantial costs. The impact of this may be reduced by the use of haplobanks and accepting a degree of human leukocyte antigen (HLA) mismatch, but this would probably necessitate the use of immunosuppressant agents, meaning that the major advantage of iPSC-derived grafts is lost (Taylor et al., 2012).

Though developments in the field of cell-based therapies have taken decades, there is much hope surrounding forthcoming trials of ESC-derived and iPSC-derived neural progenitor grafts. Increasingly, there is consideration being given to the practicalities of how these treatments may be delivered, highlighting the progress that has been made toward a useful cell-based therapy.

An alternative stem cell source that has been considered as a potential means of treating PD are bone marrow-derived mesenchymal stem cells (MSCs). These are multipotent cells that can be differentiated into tyrosine hydroxylase positive cells, which have improved motor behavior in 6-hydroxydopamine lesioned rodents (Offen et al., 2007). However, differentiation into dopaminergic neurons has been challenging, with only few published reports of this (Trzaska et al., 2007), and the utility of MSCs is likely to depend on a period of optimisation, as has been seen with iPSCs and ESCs. It has also been suggested that MSCs themselves may have neuroprotective properties through anti-inflammatory and paracrine activity, and that they may be useful as a regenerative therapy even without differentiation into dopaminergic neurons (Kim Y.J. et al., 2009; Delcroix et al., 2011). MSC grafts have been delivered to PD patients in a small open-label trial, with short-term safety demonstrated (Venkataramana et al., 2010). This study was not able to comment on whether or not there was any clinical benefit, with short follow up time, and the role of MSCs going forward remains unclear.

The main concern regarding the use of stem cell-based therapies for PD, is the theoretical potential for tumor formation. This may occur due to overgrowth of the graft, or the presence of mutations in oncogenes or tumor suppressor genes in the grafted cells. Though tumors were seen in some of the early pre-clinical trials of ESC-derived transplants, these have not been observed with improved differentiation protocols (Sonntag et al., 2007; Kikuchi et al., 2017). However, the limited follow-up time that is possible in animal models should be acknowledged, and it may be that when present for many years, as would be the case in PD patients, that these grafts do convey a risk of tumorigenesis. Furthermore, interpretation of genetic abnormalities within a graft product is challenging, and because of this, forthcoming clinical trials have adopted different strategies for genetic testing (Barker et al., 2017). Robust investigation of safety, and thorough surveillance will be vital to ensure that the risk of tumor formation is negligible, if these treatments are to be successful.

### Viral Gene-Delivery Approaches

An alternative approach toward a regenerative treatment for PD involves the use of viral vectors to achieve expression of specific genes in the striatum. These have included vectors delivering genes encoding the enzymes responsible for the production of dopamine – aromatic amine decarboxylase (AADC), tyrosine hydroxylase and cyclohydrolase-1.

Adeno-associated virus (AAV) vectors carrying AADC have been shown to result in persistent transgene expression, with consequent long-term conversion of levodopa to dopamine in non-human primates (Bankiewicz et al., 2006). Phase I trials have subsequently investigated the safety of AAV vectors delivering AADC to the putamina of PD patients (Christine et al., 2009; Muramatsu et al., 2010). Both trials reported an improvement in clinical and PET imaging parameters at 6 months, and the procedures were well tolerated. Two further phase I clinical trials conducted in PD patients using AAV vectors have been undertaken, but these have taken different therapeutic approaches. One has used a trophic factor for dopaminergic neurons (neurturin) and the other has sought to change the identity of the subthalamic output neurons from glutamatergic to glutamic acid-decarboxylase expressing ones. These trials showed initial promise, and supported the safety of gene-based therapy approaches (Kaplitt et al., 2007; Marks et al., 2008). However, randomized phase II trials were not so promising, showing no, or only mild benefit in comparison to placebo (Marks et al., 2010; LeWitt et al., 2011). AAV vectors are non-integrating vectors, so do not carry the risk of insertional mutagenesis that may occur with integrating viral vectors (Mukherjee and Thrasher, 2013). However, the main drawback of AAV vectors is that the potential size of the genetic cargo is relatively limited, meaning that the gene for only one of the enzymes in the dopamine synthesis pathway could be delivered in the aforementioned trials.

In contrast, lentivirus vectors are integrating viral vectors, with capacity for a much larger genetic cargo than AAVs – an attribute that has been exploited in viral gene-delivery trials (Palfi et al., 2014). ProSavin (Oxford Biomedica, United Kingdom) is a lentiviral product containing the genes for AADC, along with those for tyrosine hydroxylase and cyclohydrolase-1. A phase I/II trial showed that this product was well tolerated, though a significant number of patients did experience an increase in on-medication dyskinesias (Palfi et al., 2014). A further trial of a new version of ProSavin, OXB -102, is about to start in the United Kingdom and France.

## α-Synuclein as a Therapeutic Target

**FIGURE 1:**
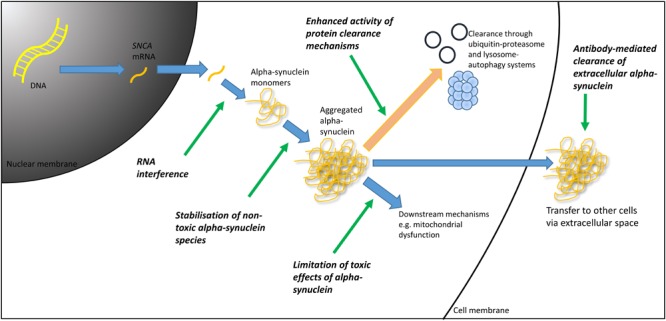
Therapeutic targets in α-synuclein mediated pathology. DNA, deoxyribonucleic acid; mRNA, messenger ribonucleic acid; RNA, ribonucleic acid.

Accumulation and aggregation of α-synuclein is the pathological hallmark of PD, and though its role is not completely understood, it appears pivotal in the pathogenesis of PD (and other α-synucleinopathies such as dementia with Lewy bodies and multiple system atrophy) (Spillantini et al., 1997). It follows therefore, that reducing the levels of pathological forms of α-synuclein may alter the course of PD. A number of experimental approaches have been investigated or are currently under investigation for their ability to potentially offer a disease-modifying effect in PD (see **Figure 1**) through targeting α-synuclein. Here, we provide an overview of some of the most promising approaches.

### Reduction of α-Synuclein Production

One mechanism by which the pathological effect of α-synuclein could be mitigated is to reduce its synthesis. This may be achieved through RNA interference technologies, in which exogenous introduction of synthetic ribonucleic acid (RNA) molecules are used to trigger selective post-transcriptional silencing of the α-synuclein gene, through messenger RNA (mRNA) degradation. Lentiviral delivery of a short-hairpin RNA (shRNA) targeting α-synuclein has been shown to silence ectopic α-synuclein expression in a rodent model and in SH-SY5Y cells, offering support to this concept (Sapru et al., 2006). Additionally, direct infusion of a small-interfering RNA (siRNA) directed against α-synuclein into the mouse hippocampus lead to a reduction in the expression of α-synuclein (Lewis et al., 2008). Following on from this, work conducted in non-human primates, in which an infusion of siRNA directed against α-synuclein was administered, demonstrated a reduction in α-synuclein mRNA, and a 40–50% reduction in α-synuclein protein levels (McCormack et al., 2010). It remains to be seen whether or not this reduction in α-synuclein levels will translate into a clinical benefit, but demonstration that these techniques have the ability to reduce α-synuclein levels is certainly promising.

Of course, one concern with suppression of α-synuclein production is that there may be negative implications due to silencing of normal α-synuclein and with this a loss of its normal physiological function. This function is not completely understood, but it appears to play a role in the docking of vacuoles to the cell membrane, so could potentially be important in neurotransmitter release (Burré et al., 2014). Reassuringly, despite a reduction in α-synuclein levels in the rodent models discussed, α-synuclein suppression did not result in any toxicity (Lewis et al., 2008). However, other studies have reported significant neurotoxicity in association with the suppression of α-synuclein including in the substantia nigra pars compacta (Robertson et al., 2004; Gorbatyuk et al., 2010; Kanaan and Manfredsson, 2012). In one of these studies, the neurodegeneration could be rescued by supplementing α-synuclein levels, suggesting that it was indeed the loss of α-synuclein that precipitated the pathology (Gorbatyuk et al., 2010). These reports highlight the need for adequate safety data if these techniques are to progress from pre-clinical to clinical studies.

Whilst these RNA-based approaches reduce translation of the α-synuclein gene, an alternative target would be to reduce transcription of the α-synuclein gene. Recently, β2-adrenergic receptor agonists (β2 agonists) including clenbuterol, have been associated with a reduction in α-synuclein gene expression (Mittal et al., 2017). Clenbuterol, a medication used in the management of asthma, was shown to reduce α-synuclein expression by 35% in a neuroblastoma cell line and in rat cortical neurons. Additionally it has the ability to cross the blood brain barrier – a necessary requirement for any of these proposed treatment approaches (Mittal et al., 2017). These preclinical trials have been supported by the results of two large Norwegian epidemiological cohorts, in which four million individuals were followed up. In these, β2 agonists were associated with a reduced risk of developing PD. Conversely, β2 antagonists (such as propranolol) were associated with an increased risk of PD (Mittal et al., 2017). Although this association requires further investigation, the prospect that β2 agonists may offer a disease-modifying effect in PD is an exciting one, particularly given the fact that there is already abundant experience with the use of these drugs for other conditions, meaning that their introduction to the clinic could be relatively quick.

### Increasing α-Synuclein Clearance

An alternative approach to targeting α-synuclein, is to enhance its clearance. This may potentially be achieved through increasing the intracellular degradation of α-synuclein through autophagy pathways and the ubiquitin-proteasome system for example, or by utilizing immune-therapies to clear extra-cellular α-synuclein, particularly given that it has been hypothesized that α-synuclein pathology may spread between cells in a prion-like fashion.

#### Immunotherapies

Active and passive immunotherapies have been investigated as a means to target and degrade extracellular α-synuclein, and have been shown to reduce α-synuclein aggregation and prevent behavioral deficits in transgenic mice (George and Brundin, 2015). Some of these treatments have reached clinical trials, with promising safety data and early results (Brundin et al., 2017).

A phase I clinical trial of an active immunotherapy vaccine (AFFITOPE PD03A, AFFiRiS, Austria) has recently been completed^1^. This is a synthetically produced vaccine containing an α-synuclein mimicking peptide. In this trial, patients with early-stage PD received repeated subcutaneous injections of AFFITOPE PD03A. Thirty-six patients were randomized to high dose or low dose AFFITOPE PD03A or placebo. A dose-dependent immune response against the peptide, as well as cross-reactivity against α-synuclein, was reported, with increasing antibody titres over time^1^. Encouragingly both doses were well tolerated and no serious drug related side effects were reported. Another one of the company’s active immunotherapies, PD01A, has been reported to be safe in early PD, over 4 years^2^.

A passive immunotherapy approach using humanized monoclonal antibodies against α-synuclein (PRX001) has also been tested. Prothena conducted a phase Ia clinical trial demonstrating a reduction in α-synuclein levels by up to 96.5% in healthy volunteers, and a subsequent phase 1b trial in PD patients in which a reduction in α-synuclein was also seen (Schenk et al., 2017). PRX001 was well tolerated and a phase II trial in patients with early stage PD is currently under way^3^.

Additional immunotherapies targeting α-synuclein are on the horizon, with the AstraZeneca and Takeda Pharmaceutical companies announcing the development of an α-synuclein antibody MEDI1341 for PD^4^. The press release regarding MEDI1341 claimed that this drug has a high affinity for the target and a reduced effector function, potentially offering a safer and more efficacious treatment, in comparison to other α-synuclein immunotherapies.

#### Enhancement of Autophagy

The proposed mechanism for the immunotherapies that have been discussed is clearance of extracellular α-synuclein, but it may also be possible to clear intra-cellular α-synuclein through other means. The lysosome-autophagy system plays an important role in α-synuclein clearance, through both chaperone-mediated autophagy and macroautophagy (Decressac et al., 2013). Enhancement of activity within this system could therefore reduce α-synuclein levels, and its propensity to accumulate and aggregate. A number of drugs have been investigated as potential means by which autophagy can be activated, including novel agents as well as the repurposing of drugs with other established indications.

Rapamycin for example, has historically been used as an immunosuppressant agent, and is a well-established inducer of macroautophagy (Moors et al., 2017), and it has been shown to reduce α-synuclein accumulation in an *in vitro* model of *GBA1* mutation-associated PD (Cullen et al., 2011). Although the unfavorable adverse effect profile of rapamycin means that is unlikely to be useful as a chronic treatment for PD, results such as this suggest that enhancing autophagy can indeed reduce total α-synuclein levels (Cullen et al., 2011; Moors et al., 2017). Several compounds have been suggested to increase autophagy activity, including for example the disaccharide trehalose (Sarkar et al., 2007; Rodríguez-Navarro et al., 2010) and the tricyclic antidepressant nortriptyline (Gassen et al., 2014), which are under consideration for entry into clinical trials.

Trehalose is of particular interest having been shown to reduce protein aggregates in models of other neurodegenerative diseases (Tanaka et al., 2004; Davies et al., 2006). Trehalose is a naturally occurring disaccharide, which appears to play an important role in stress responses in yeast (Singer and Lindquist, 1998). Whilst it has been suggested that its ability to reduce protein aggregation occurs due to a chaperone activity, or through binding and stabilization of abnormal proteins (Tanaka et al., 2004; Davies et al., 2006), it has also been shown to act via an mTOR-independent pathway to increase autophagy (Sarkar et al., 2007; Rodríguez-Navarro et al., 2010). These studies have clearly prompted interest in this compound, though a recent study of mouse primary cortical neurons found that trehalose did not prevent toxicity from exposure to α-synuclein pre-formed fibrils (Redmann et al., 2017).

With an increased understanding of the genetic basis of PD it is becoming increasingly clear that different pathogenic mechanisms may be more pronounced in certain groups of patients. It thus follows that, as our knowledge of pathological subtypes of PD expands, then different targeted therapeutic approaches may emerge. *GBA1* mutations are the most common genetic risk factor for PD (Sidransky et al., 2009). The *GBA1* gene encodes for the enzyme β-glucocerebrosidase (GCase), and homozygous mutations in this gene result in the lysosomal storage disorder, Gaucher disease (Migdalska-Richards and Schapira, 2016). Though the mechanism by which *GBA1* mutations increase the risk of PD are not fully understood, there is an established literature suggesting that dysfunction of the lysosome-autophagy system is impaired in this setting (Schöndorf et al., 2014; Bae et al., 2015; Fernandes et al., 2016). Therefore, in this group of patients, targeting autophagy may be particularly relevant. There are two clinical trials ongoing in which PD patients with *GBA1* mutations are receiving treatments aiming to correct abnormalities in the lysosomal environment. In the first, a phase II trial (MOVES-PD, Genzyme/Sanofi), patients are receiving a compound called GZ/SAR402671^5^. This inhibits the production of glycosphingolipids, which are normally catabolized by GCase, and build up in cells in PD patients with *GBA1* mutations. In the other, ambroxol, a Food and Drug Administration (FDA)-approved mucolytic, is being investigated as a potential treatment for *GBA1* mutation-associated PD (ClinicalTrials.gov Identifier: NCT02941822 and NCT02914366). Ambroxol has chaperone properties, so potentially facilitates the transit of misfolded GCase to the lysosome (Maegawa et al., 2009). In *in vitro* studies, it improves lysosomal function in dermal fibroblasts with *GBA1* mutations (Bendikov-Bar et al., 2013; McNeill et al., 2014) and increases GCase activity in non-human primates *in vivo* (Migdalska-Richards et al., 2017).

#### Repurposing of Other Drugs

Two other drugs have recently been repurposed and trialed in PD patients – the glucagon-like peptide-1 (GLP-1) agonist, exenatide, and the tyrosine kinase inhibitor, nilotinib. Exenatide is an established treatment for type two diabetes mellitus (Lovshin and Drucker, 2009), whilst nilotinib is used in the treatment of chronic myelogenous leukemia, so data already exists on the safety and tolerability of these agents in patient populations, which helped expedite their progress through clinical trials, which have shown promising results.

Exenatide has been shown to provide neuroprotective, and neurorestorative effects in toxin-based rodent models of nigrostriatal degeneration, with improvements in motor function, behavior, learning, and memory (Bertilsson et al., 2008; Kim S. et al., 2009). A recent double blinded placebo-controlled trial studied the effects of subcutaneous exenatide in patients with moderate PD (Athauda et al., 2017). The treatment was associated with positive and lasting effects on off-medication motor scores at 60 weeks. In the context of PD this follow up period is relatively short, and it remains unclear as to whether this drug reduces the progression of neurodegeneration in PD, but these early results appear promising (Athauda et al., 2017).

Nilotinib has been shown to enhance amyloid clearance, and hence has been purported as a potential means of reducing α-synuclein levels (Lonskaya et al., 2014). It acts as an inhibitor of c-abl, an oncogene involved in the regulation of cell growth, differentiation, proliferation and survival of cells. Increased levels of c-abl have been associated with PD, which is thought to result in an increase in phosphorylation and aggregation of α-synuclein (Brahmachari et al., 2016; Lindholm et al., 2016). In addition, an increase in c-abl activity leads to a reduction in the function of parkin, which is a key protein in mitochondrial biogenesis, and in which mutations result in familial PD (Lonskaya et al., 2014). Nilotinib has been shown to attenuate exogenously expressed α-synuclein levels in mice, and to reduce α-synuclein induced nigral degeneration (Hebron et al., 2013). A recent open-label study in which patients with PD with dementia or Lewy body dementia received nilotinib, demonstrated safety and tolerability of the drug in these groups, at doses of 150 and 300 mg (lower than what is used in its established indications) (Pagan et al., 2016). This study, however, involved no placebo group, and there were significant baseline differences between the two small groups, so it was not possible to comment on any clinical benefits that the drug may offer. Though the pre-clinical studies raise interesting prospects for nilotinib, there is as yet no convincing evidence of its efficacy in PD patients (Wyse et al., 2016), although another trial (NILO-PD) with this agent is currently ongoing in the United States (ClinicalTrials.gov identifier: NCT03205488).

## Conclusion

Current treatment options for PD are limited to symptomatic measures, predominantly in the form of dopaminergic medications and DBS. Though they can confer significant symptomatic benefit, they also result in troubling adverse effects, which can impair the quality of life of patients. Furthermore, none of these alter the course of disease. There is therefore a need to develop treatments that are able to restore dopaminergic tone in the striatum, in a targeted, physiological manner, and to identify drugs, which can prevent or slow ongoing neurodegeneration. A number of exciting treatment approaches are beginning to enter clinical trials. These include regenerative treatments in the form of stem cell-derived grafts and viral gene therapies designed to replace the function of the neurons that have been lost, as well as novel and repurposed drugs targeting the pathogenic mechanisms of PD, with potentially disease-modifying properties. Expanding knowledge of the pathophysiology and genetics of PD, has allowed for the development of specific treatment approaches, and it is probable that the standard treatment of PD will be dramatically altered over the coming decades, as these new treatment options emerge and are used in combination.

## Author Contributions

TS and KT contributed equally to the writing of the manuscript. RB reviewed and edited the manuscript.

## Conflict of Interest Statement

The authors declare that the research was conducted in the absence of any commercial or financial relationships that could be construed as a potential conflict of interest.

## Footnotes

1 http://www.affiris.com/news/affiris-announces-top-line-results-of-first-in-human-clinical-study-using-affitope/

2 http://www.affiris.com/news/affiris-announces-encouraging-long-term-data-from-a-series-of-first-in-human-studies-using-affitope-pd01a-targeting-oligomeric-alpha-synuclein-in-early-parkinsons-disease-patients/

3 http://ir.prothena.com/news-releases/news-release-details/prothena-announces-initiation-phase-2-pasadena-study?ReleaseID=1032188

4 https://www.astrazeneca.com/media-centre/press-releases/2017/astrazeneca-and-takeda-establish-collaboration-to-develop-and-commercialise-medi1341-for-parkinsons-disease-25082017.html

5 http://news.genzyme.com/press-release/sanofi-initiates-phase-2-clinical-trial-evaluate-therapy-genetic-form-parkinsons-disea
